# Application of an F0-based genetic assay in adult zebrafish to identify modifier genes of an inherited cardiomyopathy

**DOI:** 10.1242/dmm.049427

**Published:** 2022-06-23

**Authors:** Yonghe Ding, Mingmin Wang, Haisong Bu, Jiarong Li, Xueying Lin, Xiaolei Xu

**Affiliations:** 1 Department of Biochemistry and Molecular Biology, Mayo Clinic, Rochester, MN 55905, USA; 2 Department of Cardiovascular Medicine, Mayo Clinic, Rochester, MN 55905, USA; 3 Department of Cardiovascular Medicine, Dongzhimen Hospital, Beijing University of Chinese Medicine, Beijing 100700, China; 4 Department of Cardiothoracic Surgery, Xiangfan Hospital, Central South University, Changsha 410008, China; 5 Department of Cardiovascular Surgery, The Second Xiangfan Hospital of Central South University, Changsha 410011, China

**Keywords:** Cardiomyopathy, F0, Microhomology-mediated end joining, Modifier gene, Zebrafish, Bag3

## Abstract

Modifier genes contribute significantly to our understanding of pathophysiology in human diseases; however, effective approaches to identify modifier genes are still lacking. Here, we aim to develop a rapid F0-based genetic assay in adult zebrafish using the *bag3* gene knockout (*bag3^e2/e2^*) cardiomyopathy model as a paradigm. First, by utilizing a classic genetic breeding approach, we identified *dnajb6b* as a deleterious modifier gene for *bag3* cardiomyopathy. Next, we established an F0-based genetic assay in adult zebrafish through injection of predicted microhomology-mediated end joining (MMEJ)-inducing single guide RNA/Cas9 protein complex. We showed that effective gene knockdown is maintained in F0 adult fish, enabling recapitulation of both salutary modifying effects of the *mtor* haploinsufficiency and deleterious modifying effects of the *dnajb6b* gene on *bag3* cardiomyopathy. We finally deployed the F0-based genetic assay to screen differentially expressed genes in the *bag3* cardiomyopathy model. As a result, *myh9b* was identified as a novel modifier gene for *bag3* cardiomyopathy. Together, these data prove the feasibility of an F0 adult zebrafish-based genetic assay that can be effectively used to discover modifier genes for inherited cardiomyopathy.

## Abstract (precis)

**Summary:** Owing to modifier genes, phenotypes of many human diseases that harbor identical causal mutations can vary substantially. Using a BAG3-associated cardiomyopathy zebrafish model, we demonstrated the feasibility of a F0-based genetic assay that enables rapid discovery of disease modifiers.

## INTRODUCTION

Phenotypic variation occurs in many inherited human diseases and, as a result, has made it difficult to identify specific mechanisms and potential therapeutics. For example, patients with identical cardiomyopathy-causing mutations, such as mutations in *RBM20, TNNI3* or *BAG3*, can manifest highly variable disease onset and severity (Arad et al., 2002; Hwang et al., 2017; Norton et al., 2011; Pantou et al., 2018). Reasons for phenotypic variation are difficult to establish, as differences might result from combinatory interactions among several causative genes (Deacon et al., 2019; Gifford et al., 2019) or co-existence of a causative gene and a modifier gene(s), i.e. genes that either exacerbate or attenuate disease progression but may not cause the disease per se (Hershberger et al., 2013; Sen-Chowdhry et al., 2010). Genomic technology, such as genome-wide association studies (GWASs) in humans (Dorn, 2011; Mouton et al., 2016; Su et al., 2014; Visscher et al., 2012) and quantitative trait locus analyses in rodents (Daw et al., 2008; Le Corvoisier et al., 2003; Suzuki et al., 2002; Wheeler et al., 2005, 2009) has previously been applied to search for modifier genes. However, these statistics-based methods often end up with large genomic loci that cover many candidate genes, making it difficult to establish a precise genotype-phenotype relationship. In order to identify more-specific genetic relationships, experimental assessment requires multiple generations of breeding and a large number of mutant animals (Gifford et al., 2019), thus the demand for colony management efforts can be prohibitively high and a more-efficient genetic assay to facilitate the discovery of modifier genes would be highly desirable.

Because genetic studies in zebrafish can be carried out at a higher throughput than most other vertebrate models, zebrafish possess unique advantages when developing new methods that enable the discovery of modifier genes. Building on an efficient gene-break transposon (GBT)-based insertional mutagenesis platform, a forward genetic screen-based method has been recently established to identify modifier genes for anthracycline-induced cardiotoxicity (AIC) (Ding et al., 2013, 2016, 2011), and deleterious modifier genes, such as *dnajb6b* and *sorbs2b*, as well as salutary modifier genes, such as *rxraa* and *mtor*, were identified (Ding et al., 2019, 2016, 2020; Ma et al., 2020). The obvious advantages of this novel approach are that the identity of modifier genes can be unambiguously uncovered and that methods can be easily adjusted to the genome for systematic identification of AIC modifiers. However, it remains unverified whether this new approach based on chemically induced AIC is extendable to an inherited cardiomyopathy model that still requires the generation of a high number of double mutants.

The advent of the CRISPR/Cas9-based genome-editing technology has created new opportunities for efficient genetic studies. Several recent studies have demonstrated the feasibility of genetic analysis in F0 animals within different species, eliminating the need to generate high numbers of stable mutant lines (Ata et al., 2018; Kroll et al., 2021; Wu et al., 2018). With these new genetic tools, it is possible to rapidly screen 50 cardiomyocyte transcriptional regulators simultaneously. This breakthrough has led to the discovery of *zbtb16a* as a new gene for cardiac development (Wu et al., 2018). To ensure high knockout (KO) efficiency in F0 fish, four CRISPR/Cas9 ribonucleoprotein complexes targeting each gene of interest were injected together. While the majority of these efforts is based on the CRISPR/Cas9 platform, which induces non-homologous end joining (NHEJ), a microhomology-mediated end-joining (MMEJ)-based genome technology has been recently adapted in zebrafish embryos to faithfully recapitulate loss-of-function phenotypes in F0 animals (Ata et al., 2018; Martinez-Galvez et al., 2021). Different to NHEJ, which often leads to unpredictable DNA repair outcomes – including those that do not shift the reading frame – injection of predicted MMEJ-inducing single guide RNA (sgRNA) incurs more precise and homogenous genetic lesions while simultaneously maintaining a high level of knockout (KO) efficiency (Ata et al., 2018; Martinez-Galvez et al., 2021; Shen et al., 2018). As a consequence, injection of a single predicted MMEJ-inducing sgRNA is typically sufficient to incur predictable biallelic mutations in F0 animals (Martinez-Galvez et al., 2021).

Prompted by the success of the F0-based genetic assay in zebrafish embryos, we explored here whether this assay can achieve the same success in adult zebrafish. Therefore, we decided to further develop this assay by using *bag3^e2/e2^* KO fish, one of the first inherited cardiomyopathy models in adult zebrafish (Ding et al., 2019). *BAG3* is a defined causative gene for human dilated cardiomyopathy (DCM), encoding a co-chaperone protein that plays an important role in regulating protein quality control and autophagic proteostasis (Myers et al., 2018). Like many other inherited cardiomyopathy diseases, significant phenotypic variation has been observed among affected humans who share the same mutations in the *BAG3* gene (Domínguez et al., 2018; Norton et al., 2011). To set up such an F0-based assay, we first utilized a classic genetic breeding approach to identify common modifier genes suggested from the acquired AIC model. We found that the predicted MMEJ-based F0 genetic assay can rapidly recapitulate the modifying effects of the *bag3^e2/e2^* stable mutants. To test the robustness of the F0-based genetic assay, we analyzed eight differentially expressed genes determined in a transcriptome study of *bag3^e2/e2^* and identified *myh9b* as a new modifier gene for *bag3* cardiomyopathy. Together, our data proved the feasibility of using an F0-based genetic assay to discover modifier genes in a cardiac disease animal model.

## RESULTS

### The AIC modifier *dnajb6b* is also a modifier gene for *bag3* cardiomyopathy

**Fig. 1.**
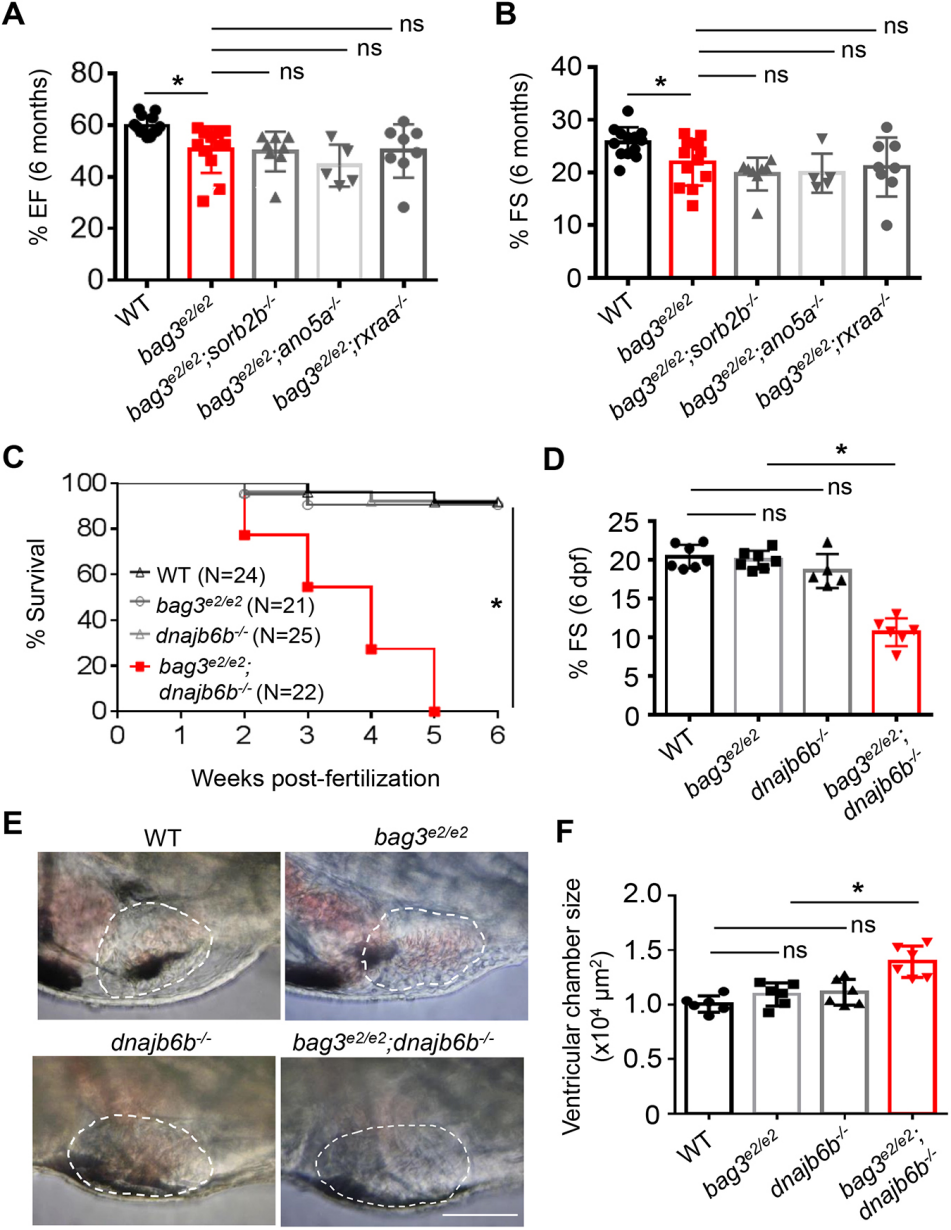
**Genetic testing identified *GBT411/dnajb6* as a deleterious modifier for *bag3* cardiomyopathy.** (A,B) Levels of ejection fraction (EF) and fractional shortening (FS) (in %) of *bag3^e2/e2^;sorbs2b^−/−^*, *bag3^e2/e2^;ano5a^−/−^* and *bag3^e2/e2^;rxraa^−/−^* double-mutant fish compared to *bag3^e2/e2^* single-mutant and WT control fish at 6 months; *n=*6-15, one-way ANOVA. (C) Survival (in %) of *bag3^e2/e2^;GB411^−/−^* double-mutant fish compared to single-mutant and WT control fish; *n=*15-24, log-rank test. (D) FS of *bag3^e2/e2^;dnajb6b^−/−^* double-mutant fish compared to single-mutant and WT control fish at 6 days dpf; *n=*6-7, one-way ANOVA. (E,F) Images of ventricular chamber outlines (E) and quantification of its size (F) of *bag3^e2/e2^;GB411^−/−^* double-mutant hearts at systolic stage compared to single mutants and WT controls, *n=*6. One-way ANOVA. Scale bar: 100 µm. *P<0.05. ns, not significant.

Because *bag3* cardiomyopathy shares common phenotypes, such as heart remodeling and cardiac dysfunction with AIC, we reasoned that some genetic modifiers of AIC identified from the forward genetic approach would exert similar modifying effects on *bag3* cardiomyopathy. To test this possibility, we took a classic genetic approach and crossed gene-break transposon (*GBT*) *002/sorbs2b, GBT136/ano5a, GBT411/dnajb6b* and *GBT419/rxraa* – four modifying mutants for AIC – into the *bag3* cardiomyopathy model (Ding et al., 2016, 2020; Ma et al., 2020). Please notice that, hereafter, we refer to these four modifying mutants by the names of their disrupted genes, i.e. *sorbs2b*, *ano5a, dnajb6b* and *rxraa*, respectively. To maximize sensitivity to any modifying effects, we generated stable double-homozygous mutants and quantified cardiac pump functions in adult fish at 6 months non-invasively by using high-frequency echocardiography (HFE). Compared to the *bag3^e2/e2^* mutant alone, we did not detect any significant changes of cardiac function in the *bag3^e2/e2^;sorb2b^−/−^, bag3^e2/e2^;ano5a^−/−^* or *bag3^e2/e2^;rxraa^−/−^* double-homozygous mutant lines (Fig. 1A,B). By contrast, *bag3^e2/e2^;dnajb6b^−/−^* fish failed to survive to adulthood, and all died within 5 weeks (Fig. 1C). Detailed studies of the *bag3^e2/e2^;dnajb6b^−/−^* double-mutant fish during embryonic stages uncovered significantly reduced cardiac function and increased ventricular chamber size, starting at 6 days post fertilization (dpf) (Fig. 1D-F). Other phenotypes include the lack of the swim bladder and an unusually protruding jaw (Fig. S1). These data suggested that *GBT411/dnajb6b,* an AIC modifying mutant, also exerts deleterious modifying effects on the *bag3^e2/e2^* cardiomyopathy.

**Fig. 2.**
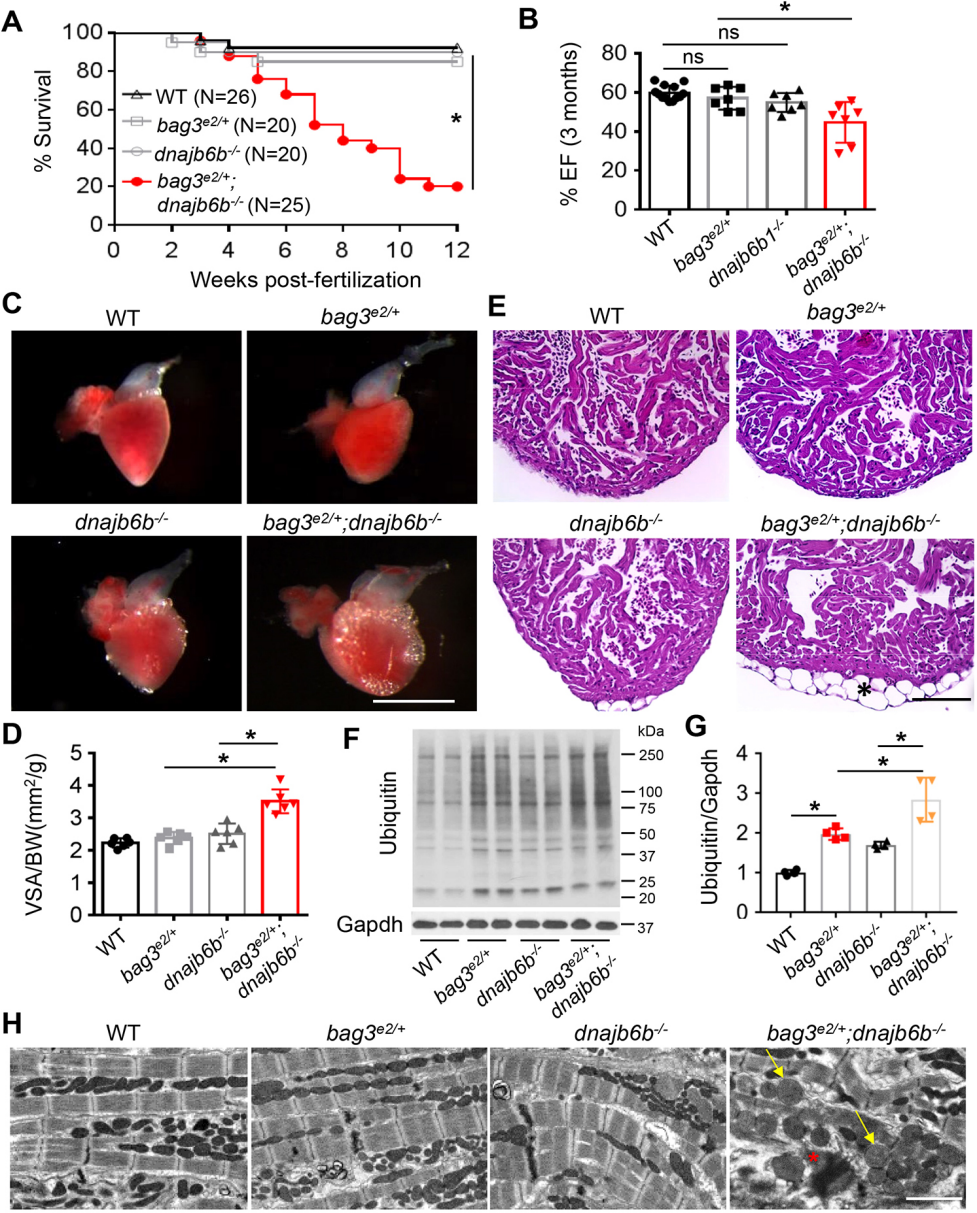
**Genetic interaction between *bag3^e2/+^* and *dnajb6b^−/−^* in adult zebrafish heart.** (A) Survival (in %) of *bag3^e/+^;GB411^−/−^* double-mutant fish compared to single-mutant and WT control fish; *n=*25-40, log-rank test. (B) Ejection fraction (EF) (in %) of *bag3^e2/+^;dnajb6b^−/−^* double mutants compared to single mutants and WT controls at 3 months post fertilization; *n=*6-8, one-way ANOVA. (C-E) Bright-field images of dissected hearts (C) show significantly enlarged ventricular surface area (VSA) in *bag3^e2/+^;dnajb6b^−/−^* double-mutant fish. VSA normalized to body weight (BW) index is plotted in D. H&E staining of heart ventricles (E) highlights the obvious lipid accumulation phenotype (asterisk); *n=*3-4, one-way ANOVA. Scale bars: 1 mm (C), 100 µM (E). (F,G) Western blot (F) and quantification analysis (G) of ubiquitylated protein levels in heart lysates of *bag3^e2/+^;dnajb6b^−/−^* double-mutant fish compared to those of single-mutant and WT control fish at 3 months; *n=*4, one-way ANOVA. (H) Confirmative TEM images, showing mitochondrial swelling (arrows) and myofibril degeneration phenotypes (asterisk) the *bag3^e2/+^;dnajb6b^−/−^* double-mutant fish hearts at 3 months. Scale bar: 2 µM. *P<0.05; ns, not significant.

We then assessed genetic interaction when these two genes are partially disrupted. Similar to the juvenile lethal phenotypes in the *bag3^e2/e2^;dnajb6b^−/−^* mutants, *bag3^e2/e2^;dnajb6b^+/−^* double-mutant fish died within 6 weeks (Fig. S2), underscoring a strong genetic interaction between *bag3* and *dnajb6b* genes. By contrast, ≤20% of *bag3^e2/+^;dnajb6b^−/−^* double-mutant fish were viable for at least 12 weeks (Fig. 2A), enabling characterization of adult phenotypes that were the result of concomitant deficiency of *bag3* and *dnajb6b* genes. Significantly declined cardiac function, accompanied by enlarged ventricular surface area (VSA) was observed in *bag3^e2/+^;dnajb6b^−/−^* double-mutant fish at 3 months (Fig. 2B,C). At this age, these fish displayed a ‘protruding jaw’ phenotype, similar to that observed in *bag3^e2/e2^;dnajb6b^−/−^* double-homozygous embryos at 6 dpf (Fig. S3). In the *dnajb6b^−/−^* mutant, we also noticed mild lipid accumulation on the surface of the ventricular chamber and elevated protein ubiquitylation, both of which are exaggerated in the hearts of *bag3^e2/+^;dnajb6b^−/−^* double mutant fish (Fig. 2D,E). In addition, by using transmission electron microscopy (TEM) analysis, we observed abnormal mitochondria swelling and myofibril loss in the hearts of *bag3^e2/+^;dnajb6b^−/−^* double mutants but not in those of single mutants (Fig. 2F). Together, these data confirmed a genetic interaction between *dnajb6b* and *bag3* genes in cardiomyopathy. Moreover, our data suggested functions of *bag3*−*dnajb6b* in the regulation of lipid metabolism and proteostasis, two pathological events that are observed in cardiomyopathy (Goldberg et al., 2018; Henning and Brundel, 2017; Wende and Abel, 2010).

Mouse Dnajb6(S), a predominantly somite-expressed short isoform of Dnajb6, has been shown to directly bind Bag3 protein in the skeletal muscle (Sarparanta et al., 2012). The *GBT411/dnajb6b* mutant fish harbor an insertion that is located after the exons encoding the short isoform and before the exons encoding the long isoform, thus specifically disrupts DNAJB6(L), a cardiac-enriched long isoform of DNAJB6 (Ding et al., 2016). We, thus, enquired whether DNAJB6(L) also binds the BAG3 protein in humans. By using an *in vitro* protein pulldown assay with human HEK293 cells, we confirmed binding between human DNAJB6(S) and human BAG3 (Fig. S4A). Moreover, we also noticed physical interaction between DNAJB6(L) and BAG3 (Fig. S4A), and that human BAG3 and zebrafish Dnajb6b proteins colocalize within the sarcomere, as reflected by the human BAG3-EGFP fusion protein and the RFP reporter within the *GBT411/dnajb6b* zebrafish line, respectively (Fig. S4B). Taken together, our biochemical and colocalization studies both supported a genetic interaction between *dnajb6b* and *bag3* in the zebrafish heart.

### Injection of a predicted MMEJ-inducing sgRNA in F0 adult zebrafish recapitulated salutary modifying effects of *mtor* and deleterious modifying effects of *dnajb6b* on *bag3* cardiomyopathy

To accelerate the identification of modifiers, we investigated whether the predicted MMEJ-based F0 assays can be used in *bag3* cardiomyopathy adult zebrafish models. We initially tested the *tyrosinase* (*tyr*) gene, since loss-of-function of *tyr* manifests a loss-of-melanophore phenotype that is easily scored non-invasively (Ata et al., 2018). Indeed, we found that fish embryos injected with a high dose – i.e. 5 µM – of the predicted MMEJ-inducing *tyr* sgRNA (Ata et al., 2018) exhibited near-complete loss of pigmentation at 3 dpf. At 3 months, they also manifested near-complete loss of pigmentation (Fig. S5A) and a KO score maintained at ∼85%, as obtained from tail fin analysis (Fig. S5B). In contrast, a low dose i.e. 1 µM – of *tyr* sgRNA yielded lower KO scores and an increase in pigmentation (Fig. S5A,B). Thus, different doses of injected *tyr* sgRNA correlated with the KO score levels as well as the severity of pigmentation phenotype. In addition, the KO scores between heart and tail fin, or kidney and tail fin were highly correlated (R^2^ (coefficient of determination) = 0.94 and 0.96, respectively) (Fig. S5C), suggesting that the tail fin can be used as a representative to measure genetic deficiency in internal organs. Taken together, this pilot analysis of a predicted MMEJ-inducing *tyr* sgRNA strongly suggested the feasibility of rapid genotype–phenotype correlation in F0 adult zebrafish.

**Fig. 3.**
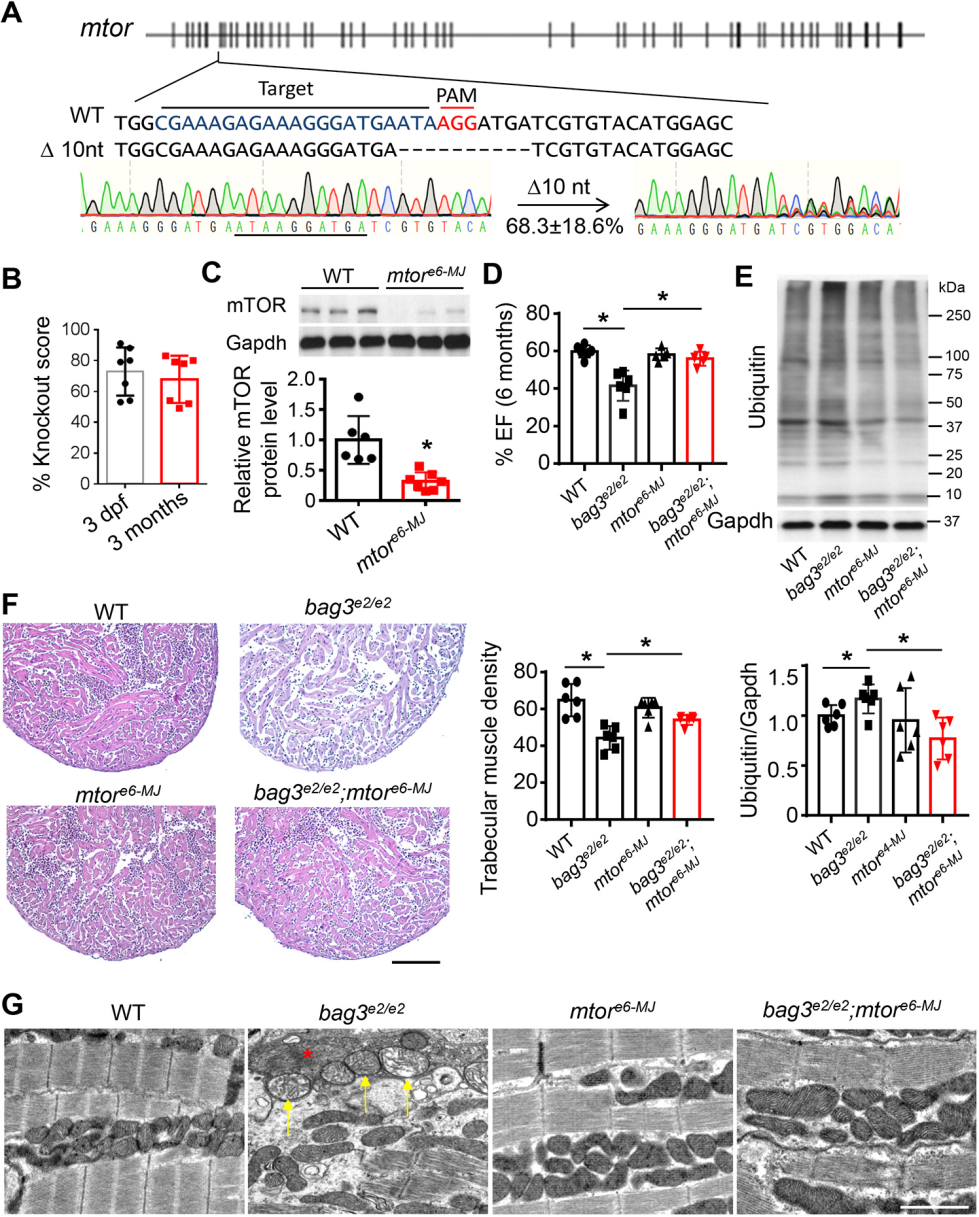
**Injection of the predicted MMEJ-inducing *mtor*-sgRNA exerted salutary modifier effects on *bag3* cardiomyopathy at F0.** (A) Design of the predicted MMEJ-inducing sgRNA sequence targeted to the exon 6 within the *mtor* gene (*mtor^e6-MJ^*). Injection of *mtor^e6-MJ^* sgRNA generated frame-shift mutant alleles, with 68.3% predicted to result from microhomology recombination (10-nucleotide deletion). (B) The *mtor* gene KO score (in %) in *mtor^e6-MJ^*-injected fish embryos at 3 dpf. (C) Western blotting and quantification analysis of mTOR protein in the *mtor^e6-MJ^* fish compared to non-injected WT controls at 3 dpf; *n=*6, one way-ANOVA. (D) Ejection fraction (EF) of *the mtor^e6-MJ^*-injected *bag3^e2/e2^* fish (*mtor^e6-MJ^*;*bag3^e2/e2^*) compared to *bag3^e2/e2^* mutant alone, *mtor^e6-MJ^* and non-injected WT controls at 6 months; *n=*6, one-way ANOVA. (E) Western blot (top panel) and quantification analysis (below) of ubiquitylated protein levels in *mtor^e6-MJ^*-injected *bag3^e2/e2^* fish hearts compared to those non-injected controls at 6 months; *n=*6, one-way ANOVA. (F) Representative H&E staining images of the apex area (left panel) and quantification of the trabecular muscle density (right panel) from the *mtor^e6-MJ^*;*bag3^e2/e2^* fish compared to those of corresponding controls at 6 months; *n=*6, one-way ANOVA. Scale bar: 100 µM. (G) Confirmative TEM images of *mtor^e6-MJ^*-injected *bag3^e2/e2^* fish hearts (*bag3^e2/e2^*;*mtor^e6−MJ^*) compared to those of corresponding controls (WT, *bag3^e2/e2^* and *mtor^e6−MJ^*) demonstrate largely restored mitochondrial swelling (arrows) and myofibril degeneration (asterisk) in the *mtor^e6-MJ^*-injected *bag3^e2/e2^* fish at 6 months. Scale bar: 2 µM; *P<0.05.

To confirm whether predicted MMEJ-based genome editing technology is faithful for assessing genetic interactions in F0 adult fish, we decided to target the *mtor* gene because *mtor* haploinsufficiency was recently reported to exert therapeutic effects on *bag3* cardiomyopathy (Ding et al., 2019). We designed a predicted MMEJ-inducing sgRNA by using the Microhomology-mediated End joining kNockout Target Heuristic Utility (MENTHU) tool (http://genesculpt.org/menthu/) and targeted at the 6th exon of the *mtor* gene. Injection of the sgRNA resulted in a mixture of genetic lesions. As revealed by Tracking Indels by DEcomposition (TIDE) analysis (Brinkman and van Steensel, 2019), the average frame-shift KO score is ∼72.9% in injected embryos, which was maintained at ∼67.9% at 3 months, 68.3% of which harbors a 10-nucleotide deletion allele predicted to result from microhomology recombination (Fig. 3A,B). mTOR protein levels decreased to ∼30% of that in non-injected controls (Fig. 3C). We then injected this *mtor* sgRNA (hereafter referred to as *mtor^e6−MJ^*) into embryos derived by *bag3^e2/+^* incrossing, which were raised for up to 6 months, and identified the *mtor^e6−MJ^*-injected *bag3^e2/e2^* double mutants (hereafter referred to as *bag3^e2/e2^*;*mtor^e6−MJ^*). We noticed that ejection fraction (EF) in the *bag3^e2/e2^*;*mtor^e6−MJ^* fish was significantly rescued compared to that in the *bag3^e2/e2^* cardiomyopathy model (Fig. 3D). Additionally, we detected a reduction in levels of ubiquitylated protein, increased trabecular muscle density, and partially restored sarcomeric and mitochondrial swelling defects in *bag3^e2/e2^*;*mtor^e6−MJ^* fish (Fig. 3E-G). Together, these results demonstrated that the F0-based genetic assay largely recapitulates salutary modifier effects of the *mtor* haploinsufficiency on *bag3* cardiomyopathy.

**Fig. 4.**
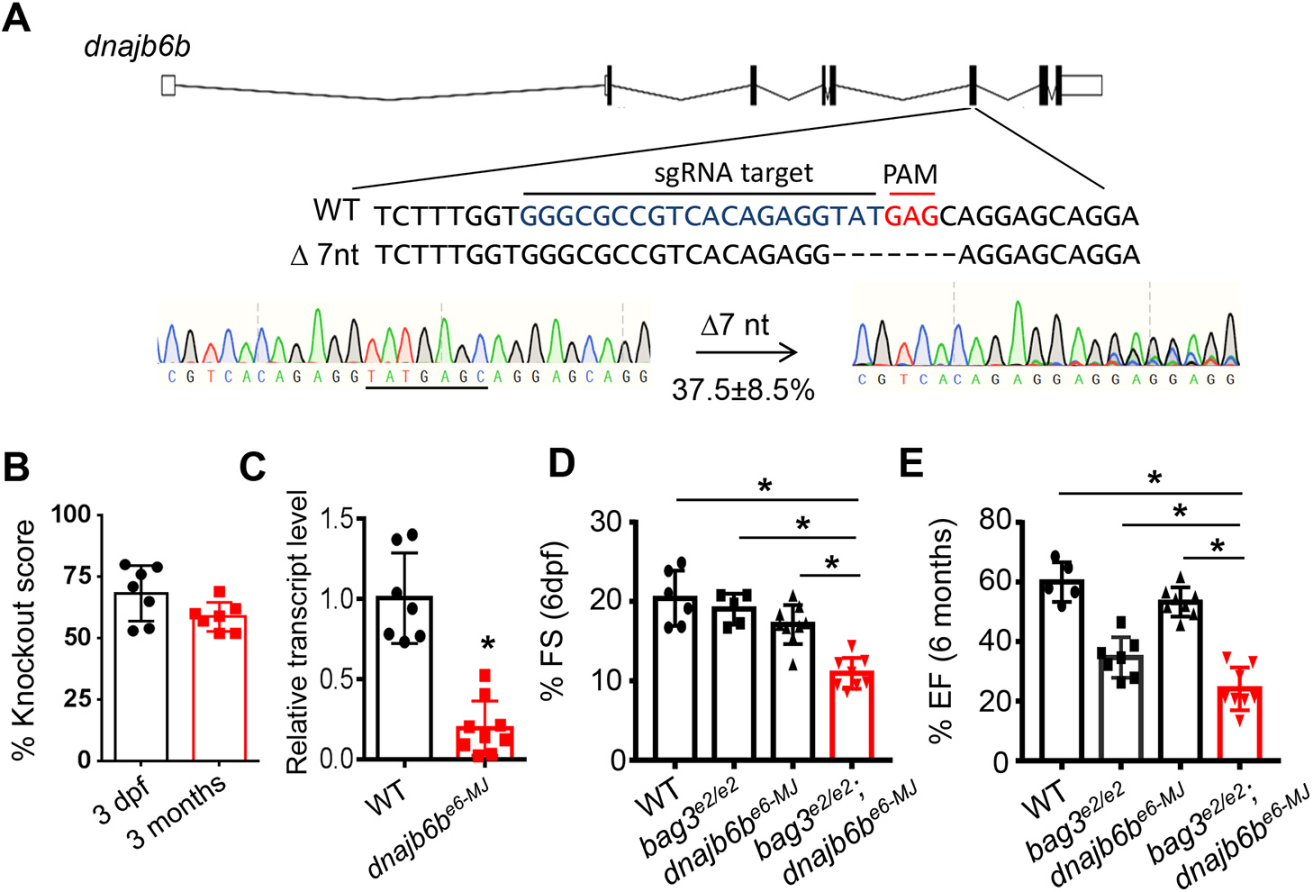
**Injection of the predicted MMEJ-inducing *dnajb6b*-sgRNA into *bag3^e2/e2^* F0 fish manifested severe cardiac dysfunction.** (A) Design of the predicted MMEJ-based sgRNA sequence targeting the exon 6 of the *dnajb6b* gene (*dnajb6b^e6-MJ^*). Injection of *dnajb6b^e6-MJ^* into bag3^e2/e2^ fish (*bag3^e2/e2^*;*dnajb6b^e6−MJ^*) generated frame-shift mutant alleles, with 37.5% predicted to result from microhomology recombination (7-nucleotide deletion). (B) Plotted is the *dnajb6b* gene knockout (KO) score (in %) of *bag3^e2/e2^*;*dnajb6b^e6−MJ^* embryos at 3 dpf and fish at 3 months. (C) Transcript levels of *dnajb6b* in hearts of *bag3^e2/e2^*;*dnajb6b^e6−MJ^* fish compared to those of non-injected controls at 3 months; *n=*10. Unpaired Student’s *t*-test. (D,E) Fraction shortening (FS) and ejection fraction (EF) in *bag3^e2/e2^*;*dnajb6b^e6−MJ^* fish compared to that in *bag3^e2/e2^* mutant, *dnajb6b^e6-MJ^* or non-injected WT control fish at 6 dpf (embryonic) and 6 months (adult). *n=*5-8, one-way ANOVA.

**Fig. 5.**
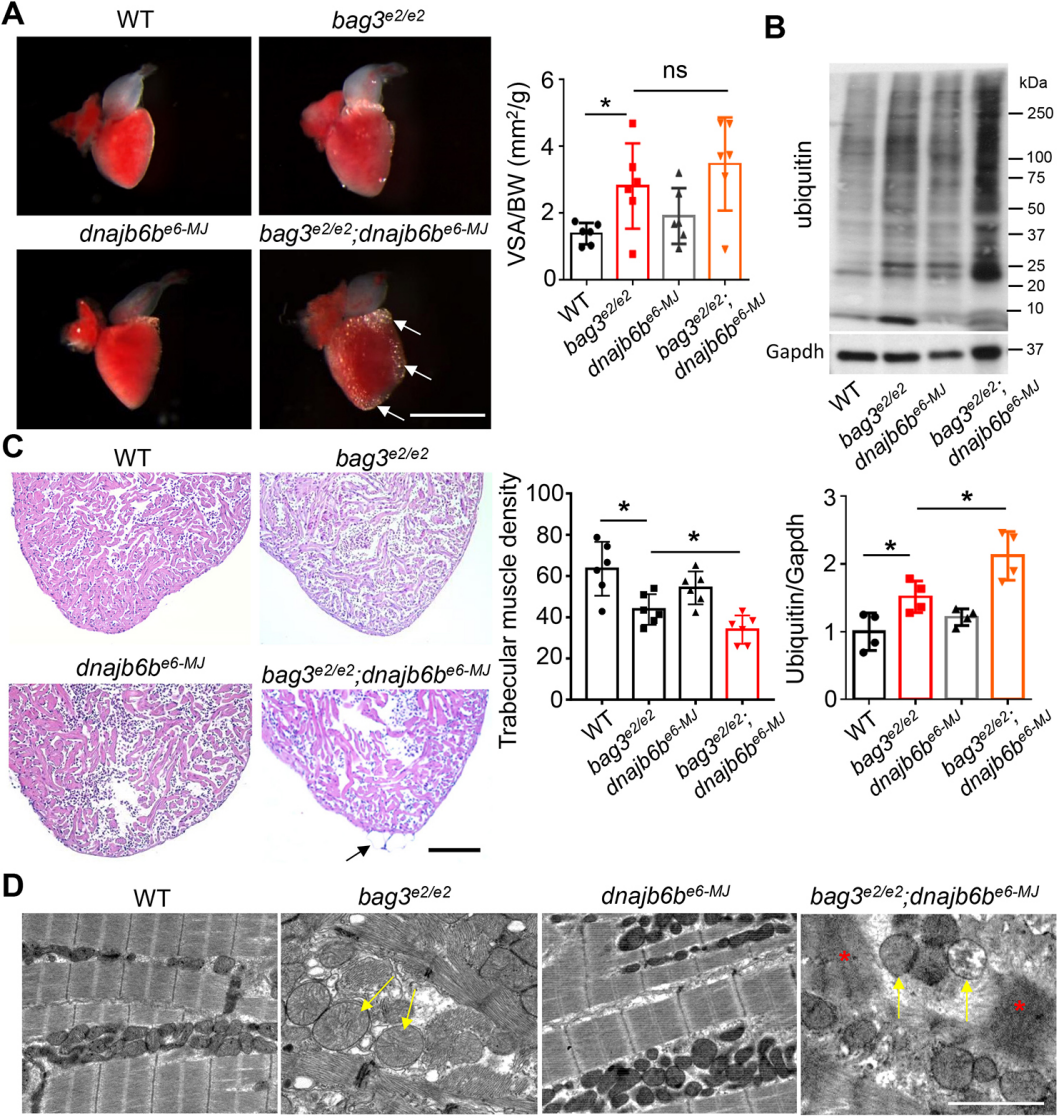
**The predicted MMEJ-inducing *dnajb6b*-sgRNA-injected *bag3^e2/e2^* F0 adult fish manifested several phenotypic traits that are similar to those in stable *bag3^e2/+^;dnajb6b^−/−^* double mutants.** (A) Bright-field images of hearts (left panel) and bar graph showing ventricular surface area (VSA) normalized to body weight (BW) (right panel) of fish as indicated. *dnajb6b^e6-MJ^*;*bag3^e2/e2^* fish exhibited lipid accumulation in the heart (arrows), as well as enlarged VSA/BW levels; *n=*6, one-way ANOVA. Scale bar: 1 mm. (B) Western blot (top panel) and quantification analysis of ubiquitylated protein levels (bottom panel) in the *dnajb6b^e6-MJ^;bag3^e2/e2^* fish hearts compared to *bag3^e2/e2^* mutant alone, *dnajb6b^e6-MJ^* or non-injected WT controls at 6 months; *n=*4, one-way ANOVA. (C) Representative H&E staining of the apex area in fish as indicated (left panel) and quantification of the trabecular muscle density (right panel) in hearts of *dnajb6b^e6-MJ^*;*bag3^e2/e2^* fish at 6 months compared to that in corresponding controls. The arrow points to lipid droplets; *n=*6, one-way ANOVA. Scale bar: 100 µM. (D) Confirmative TEM images of *dnajb6b^e6-MJ^*;*bag3^e2/e2^* fish hearts compared to those of corresponding controls demonstrate severe mitochondrial swelling (arrows) and myofibril degeneration (asterisks) phenotypes that are similar to those detected in hearts of *bag3^e2/+^;dnajb6b^−/−^* double-mutant fish. Scale bar: 2 µM; *P<0.05. ns, not significant.

Next, we tested whether injection of a predicted *dnajb6b* MMEJ-inducing sgRNA would recapitulate modifying effects between *bag3* and *dnajb6* genes in F0 fish. We designed a predicted MMEJ-inducing sgRNA targeting the *dnajb6b* gene at exon 6, mimicking the RP2 transposon insertional position in the *GBT411/dnajb6b* mutant (Fig. 4A) (Ding et al., 2013). Injection of *dnajb6b* sgRNA (hereafter referred to as *dnajb6b^e6−MJ^*) resulted in genetic lesions in the targeted locus with an average frame-shift KO score of 68.3% in F0 embryos. The frame-shift KO score was maintained at ∼58.7% in adult fish, among which 37.5% were the predicted 7-nucleotide deletion alleles presumably resulting from microhomology recombination (Fig. 4A,B). At RNA level, injection of *dnajb6b^e6−MJ^* resulted in reduction of *dnajb6b* transcripts to ∼19% compared to that in non-injected controls (Fig. 4C), probably because of nonsense-mediated decay. We refer to F0 *bag3^e2/e2^* fish injected with a *dnajb6b* sgRNA as *bag3^e2/e2^*;*dnajb6b^e6−MJ^* fish. Compared to non-injected *bag3^e2/e2^* controls, *bag3^e2/e2^*;*dnajb6b^e6−MJ^* fish exhibited significantly reduced fraction shortening (FS) and EF (Fig. 4D,E). Interestingly, further detailed phenotyping of the F0 *bag3^e2/e2^;dnajb6b^e6−MJ^* fish identified defects that were similar to those of the stable *bag3^e2/+^;dnajb6b^−/−^* double mutant, including increased lipid deposits on the ventricular surface, elevated ubiquitylated protein levels, reduced ventricle trabecular muscle density, abnormally swollen mitochondria and myofibril loss (Fig. 5). Together, these data suggest that injection of a predicted MMEJ-inducing sgRNA targeting *dnajb6b* was able to recapitulate many but not all modifying effects of *GBT411/dnajb6b* on *bag3* cardiomyopathy.

### Screening of eight differentially expressed genes by using the F0 genetic assay identified *myh9b* as a new modifier gene for *bag3* cardiomyopathy

**Fig. 6.**
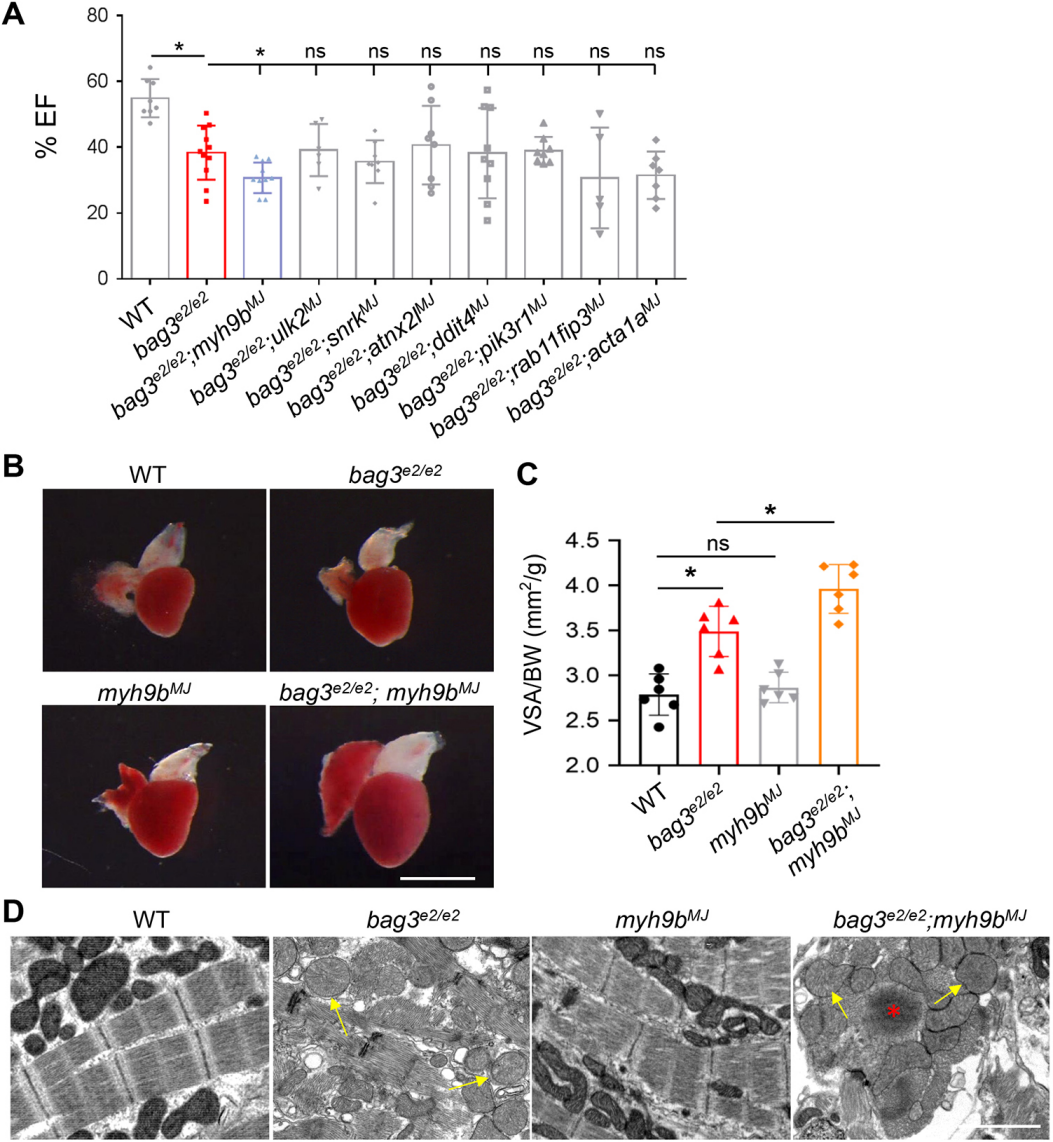
**Identification of *myh9b* as a new deleterious modifier gene for *bag3* cardiomyopathy through predicted MMEJ-based genome editing at F0.** (A) Ejection fraction (EF) of *bag3^e2/e2^* fish at 6 months after injection with each of the eight predicted MMEJ-based sgRNAs targeting eight differentially expressed proteostasis/autophagy genes compared to *bag3^e2/e2^* single-mutant and WT control fish at 6 months; *n=*4-11, one-way ANOVA. (B,C) Bright-field images of hearts (B) and bar graph (C) showing ventricular surface area (VSA) normalized to body weight (BW) in fish as indicated. The *myh9b^MJ^*-injected *bag3^e2/e2^* fish (*bag3^e2/e2^*;*myh9b^MJ^*) exhibited increased levels of VSA/BW compared to that in the *bag3^e2/e2^* mutant alone, *myh9b^MJ^* or non-injected WT controls at 6 months; *n=*6, one-way ANOVA. Scale bar: 1 mm. (D) Confirmative TEM images to compare hearts of *bag3^e2/e2^*;*myh9b^MJ^* fish to those of *bag3^e2/e2^* mutant alone, *myh9b^MJ^* or non-injected WT controls at 6 months. Arrows indicate mitochondrial swelling; the asterisk indicates sarcomeric *Z*-disc aggregation. Scale bar: 2 µM; *P<0.05, ns, not significant.

Encouraged by our success in recapitulating two known modifier genes for *bag3* cardiomyopathy, we then enquired whether the predicted MMEJ-based genetic interaction assay in F0 fish is sufficiently robust for discovering new modifier genes. Our previous RNA-seq analysis of heart tissues identified a number of differentially expressed genes between the *bag3^e2/e2^* homozygous fish and WT sibling controls (Ding et al., 2019). We focused on differentially expressed genes involved in proteostasis/autophagy because BAG3 is a proteostatic protein and dysregulated autophagy had been found in both *bag3^e2/e2^* fish and *Bag3* conditional KO mouse hearts (Fang et al., 2017; Ruparelia et al., 2020). Of 27 differentially expressed genes previously obtained by RNA-seq analysis, eight – including *myh9b, ulk2a, snrkb, atxn2 l, ddit4, pik3r1, rab11fib3* and *acta1a* – were validated experimentally by using quantitative reverse transcription polymerase chain reaction (qRT-PCR) analysis (Fig. S6). We designed predicted MMEJ-inducing sgRNAs targeting these genes and obtained >50% KO score in seven out of eight genes (Table 1). Individual sgRNAs were then injected into the offspring of *bag3^e2/+^* incrossed fish embryos. We found that injection of the sgRNA targeting at the 12th exon of *myh9b* gene exaggerates *bag3* cardiomyopathy, as indicated by significantly reduced EF and enlarged heart chamber size in the *bag3^e2/e2^;myh9b^MJ^* fish compared to non-injected *bag3^e2/e2^* controls (Fig. 6A-C). In contrast, the other seven predicted MMEJ-inducing sgRNAs did not cause any significant changes of EF in *bag3^e2/e2^* fish at 6 months of age (Fig. 6A). Additionally, TEM analysis discovered severe mitochondrial swelling and sarcomeric disarray phenotypes in *bag3^e2/e2^;myh9b^MJ^* fish but not in fish injected with *myh9b^MJ^* alone (Fig. 6D).

**Table 1.**
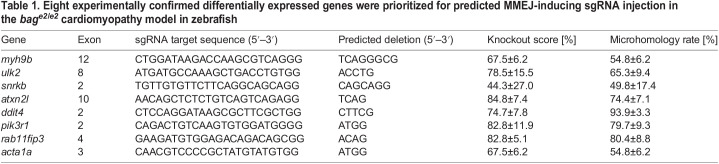
Eight experimentally confirmed differentially expressed genes were prioritized for predicted MMEJ-inducing sgRNA injection in the *bag^e2/e2^* cardiomyopathy model in zebrafish

**Fig. 7.**
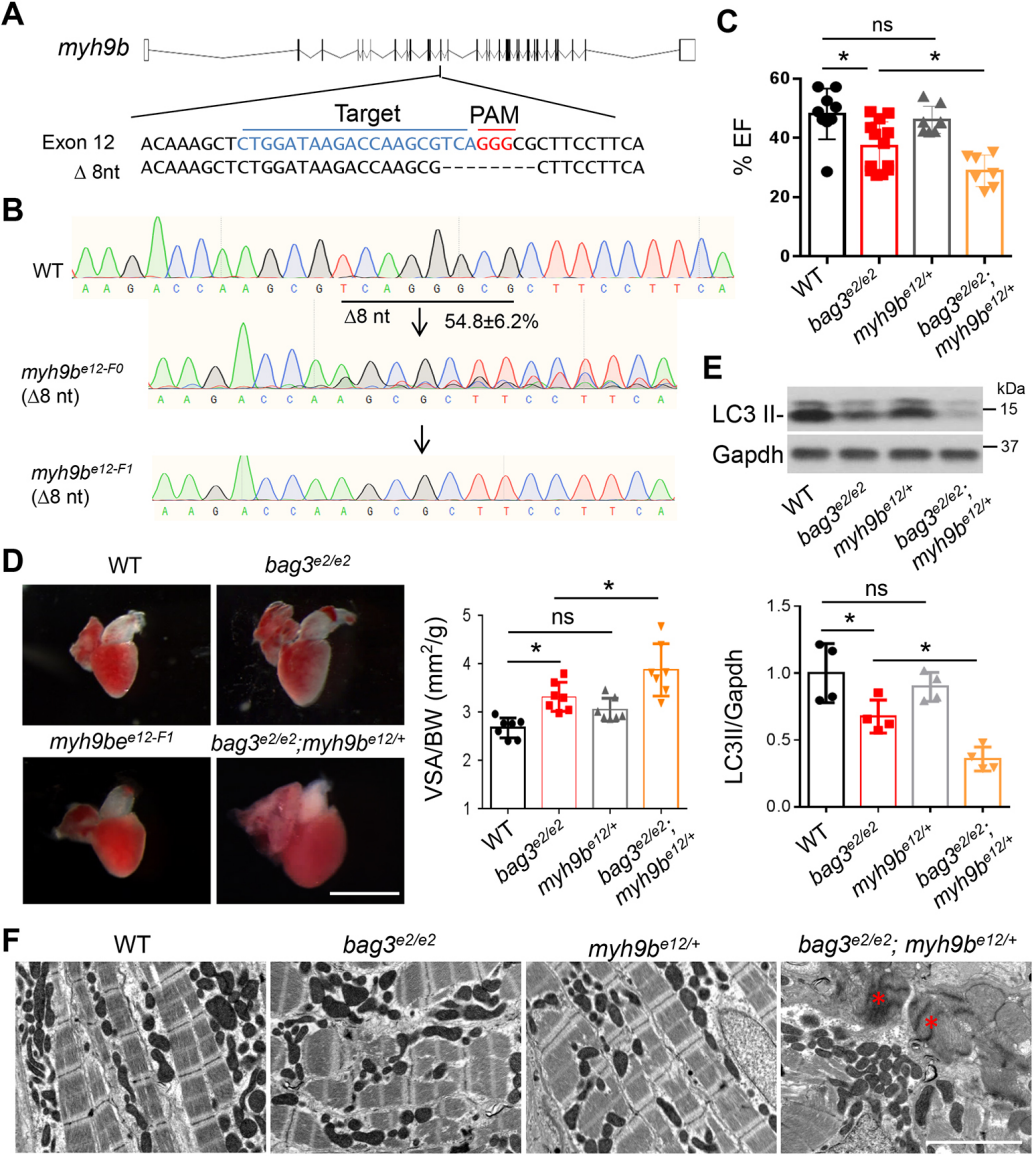
**The stable *myh9b* heterozygous mutant deteriorated *bag3* cardiomyopathy at adult stage.** (A) Schematic of the predicted MMEJ-inducing *myh9b* genetic lesion of 8-nucleotides generated by injection of an sgRNA targeting sequences within the 12th exon. Dashed lines indicate an 8-nucleotide-long deletion. (B) Chromographs illustrating the sequences of the wild-type *myh9b* and the mutant alleles with the predicted 8-nucleotide-long deletion in F0 and F1 fish. (C) Quantification of cardiac function. Ejection fraction (EF) (in %) measured by using echocardiography in *bag3^e2/e2^;myh9b^e12/+^* double-mutant fish compared to single-mutant and WT control fish at 6 months; *n=*7-11, one-way ANOVA. (D) Left panel: Representative images of isolated hearts from fish as indicated. Right panel: Quantification of ventricular surface area (VSA) normalized to the body weight (BW) of fish at 6 months; *n=*7, one-way ANOVA. (E) Western blotting (top) and quantification LC3 II protein levels (bottom) of hearts from *bag3^e2/e2^*;*myh9b^e12/+^* double-mutant fish, *bag3^e2/e2^*, *myh9b^e12/+^* or WT control fish at 6 months. Levels of Gapdh were used as control. *n=*4, one-way ANOVA. (F) TEM images of *bag3^e2/e2^*;*myh9b^e12/+^* double-mutant fish hearts, *bag3^e2/e2^*, *myh9b^e12/+^* and WT controls at 6 months. Asterisks indicate *Z*-disc aggregation. Scale bar: 2 µM (D). *P<0.05. ns, not significant.

To confirm the modifying effects of *myh9b* gene on *bag3* cardiomyopathy, we incrossed the *bag3^e2/+^;myh9b^MJ^* F0 fish with high KO scores to generate stable *bag3^e2/e2^;myh9b*^e12/+^ mutants at F1. The myh9b F0 fish comprised a mix of different genetic lesions with averaged KO scores of 67.5%, among which 54.8% indels are 8-nucleotide-long deletion alleles were acquired by predicted microhomology recombination (Fig. 7A and Table 1). By contrast, the *myh9b* homozygous embryos (*myh9b^e12/e12^*) at F1 derived from F0 incrosses harbored exclusively the 8-nucleotide-long deletion allele, as evidenced by Sanger sequencing (Fig. 7A,B). As expected, KO scores for all *myh9b* F1 heterozygous mutant fish were ∼50%. Although we failed to recover the *myh9b^e12/e12^* fish to adulthood – probably because of a recessive lethal phenotype – we obtained *myh9b^e12/+^*, *^ ^*which remained viable at the adult stage. We further obtained age-matched groups consisting of *bag3^e2/e2^*, *bag3^e2/e2^;myh9b^e12/+^* and corresponding WT sibling controls from genetic crossing followed by genotyping PCR. Whereas the *bag3^e2/e2^* cardiomyopathy model manifested reduced EF, the stable *bag3^e2/e2^;myh9b^e12/+^* double-mutant fish exhibited further reduced EF compared to WT fish (Fig. 7C). Similar to the *bag3^e2/e2^;myh9b^MJ^* F0 fish, the stable *bag3^e2/e2^;myh9b^e12/+^* mutant F1 fish also displayed more-enlarged ventricular chamber size, further reduced autophagy level and increased disarray of the sarcomere (Fig. 7D,E; Fig. S7). Together, these data confirmed *myh9b* as a new modifier gene for *bag3* cardiomyopathy.

## DISCUSSION

### A predicted MMEJ-inducing sgRNA injection approach in F0 adult zebrafish can be used to discover genetic modifiers of an inherited cardiomyopathy

Prompted by the predicted MMEJ-induced genome editing technology that has recently been established in zebrafish F0 embryos for functional genomic studies (Ata et al., 2018; Martinez-Galvez et al., 2021), we present here several pieces of evidence to demonstrate an expanded application of this more-precise gene editing technology in F0 adult fish. First, similar to a recent report (Wu et al., 2018), we showed that injection of a predicted MMEJ-inducing sgRNA targeting the *tyr* gene incurred a loss-of-melanophore phenotype that is sustained from embryonic to adult stage in F0 animals. Second, we demonstrated that modifying effects of *dnajb6* and *mtor* on the *bag3* cardiomyopathy can be largely recapitulated in F0 adult fish. Last, we utilized this F0-based genetic platform to screen differentially expressed genes and identified *myh9b* as a new genetic modifier for *bag3* cardiomyopathy. This finding was subsequently confirmed by the stable *bag3^e2/e2^;myh9b^e12/+^* double mutant. Together, these data indicate that effective gene knockdown in adult F0 animals can be generated by injection of a predicted MMEJ-inducing sgRNA, enabling reliable establishment of genotype-phenotype relationship. The success of this genetic technology platform effectively minimalizes the need of multi-generation genetic crossing to assess genetic modifying effects, representing a rapid approach for discovering modifier genes for an adult-onset disease, such as cardiomyopathy.

The use of MENTHU, a double-strand-break repair-prediction algorithm, helped to identify sgRNAs with a predictable genetic lesion with high knockdown efficiency. Based on TIDE analysis, sgRNAs for eight differentially expressed genes induced a predicted microhomology allele in the targeted loci at rates ranging from 49.8% to 93.9% among all eight genetic lesions in F0 adult fish (Table 1). Seven out of eight targeted loci incurred >50% KO score upon the first try. Of note, we used a single predicted MMEJ-inducing sgRNA for each gene, which is different to recent F0-based embryonic studies that have used NHEJ-inducing sgRNAs, and in which 3-4 sgRNAs targeting the same gene were injected together (Hoshijima et al., 2019; Kroll et al., 2021; Wu et al., 2018). Because high KO efficiency is needed in embryonic studies to generate biallelic alleles that recapitulate stable homozygous mutants, co-injection of multiple sgRNAs effectively overcomes the confounding factor that approximately one-third of indels induced by each sgRNA do not shift the reading frame. By contrast, assessment of modifier effects in adult zebrafish does not require extremely high KO scores, i.e. a KO score of ∼50% would be sufficient. Importantly, the ratio of non-reading frame shifting indels would be much lower in fish injected with predicted MMEJ-inducing sgRNAs than in those injected with NHEJ-inducing sgRNAs.

We acknowledge several shortcomings of this F0-based genetic assay, most of which are associated with the complicated nature of genetic lesions. First, unlike a stable mutant that comprises a single genetic lesion, each F0 fish harbors a mixture of different genetic lesions in the same genomic locus, including those that do not shift the reading frame. This is why we favor the predicted MMEJ-inducing sgRNAs over normal CRISPR sgRNAs, i.e. the majority of genetic lesions are the predicted deletion alleles that shift the reading frame. Second, phenotypes in stable double mutants might not be fully recapitulated in F0 mutants. For example, the *bag3^e2/e2^*;*dnajb6b^e6−MJ^* double mutants manifested milder phenotypes than either *bag3^e2/e2^;dnajb6b^−/−^* or *bag3^e2/e2^; dnajb6b^+/−^* double mutants, both of which died within 5 weeks. Although *bag3^e2/e2^*;*dnajb6b^e6−MJ^* recapitulated several adult cardiac phenotypes in the *bag3^e2/+^;dnajb6b^−/−^* double-mutant fish (Fig. 4), it did not recapitulate the jaw-protruding phenotype. We postulated that one potential reason is that an F0 fish with a 50% KO score does not fully recapitulate a stable heterozygous mutant, whereby all cells contain a genetic lesion in one of the two alleles. Instead, cells in F0 fish might be a mixture of cells without genetic lesion, with a single genetic lesion and with biallelic genetic lesions. Another potential reason is the different functional consequence between the F0*dnajb6b^e6−MJ^* and stable *dnajb6b^+/−^* on *bag3* cardiomyopathy. Whereas the anticipated genetic lesions incurred by the predicted MMEJ-inducing sgRNA presumably lead to truncated peptides and is of loss-of-function nature, more experimental evidence is needed. Third, because phenotypic assays were performed in the F0 generation, off-target effects of the sgRNA might exist and might confound phenotyping interpretation. However, despite these shortcomings, genotype-phenotype relationships can still be largely established in adult fish, as indicated by the three types of experimental evidence summarized earlier in the discussion. To ultimately address these limitations, we strongly recommend the confirmation of candidate genetic interactions by generating stable double mutants, which are available at F1.

### Genetic modifiers suggest a pathophysiology of inherited cardiomyopathy

In this article, we initially identified the modifying effects of *dnajb6b* gene deficiency on *bag3* cardiomyopathy by using classic genetic analysis. Together with *mtor* as another common genetic modifier of *bag3* cardiomyopathy and AIC, our data support the concept that cardiomyopathies of different etiology do share common pathological pathways (Tadros et al., 2021). Thus, one plausible future strategy to discover genetic modifiers of inherited cardiomyopathy would be to test candidate genetic modifiers of AIC, which can be obtained by large-scale forward genetic screens in zebrafish.

Identification of new modifier genes for a human disease are helpful to unveil new pathophysiologic mechanisms, aiding the development of disease intervention. For example, the discovery of disrupted *dnajb6b* function as a deleterious modifier for AIC suggested unfolded protein response (UPR) as the downstream signaling event and overexpression of *dnajb6b* in cardiomyocytes as a therapeutic avenue (Ding et al., 2016). Discovery of *rxraa* as a salutary modifier for AIC suggested defective endothelial barrier function as a pathological event in the early phase of AIC and activation of retinoic acid (RA) signaling as a therapeutic avenue (Ding et al., 2016; Ma et al., 2020). Here, we unveiled the modifying effects of *dnajb6b* deficiency on *bag3* cardiomyopathy. Because *dnajb6b* encodes a co-chaperone protein that facilitates degradation of misfolded proteins (Hageman et al., 2010) and because increased aggregation of ubiquitylated proteins has been detected in the *bag3;dnajb6b* co-deficient fish (Fig. 5B), we speculated that Dnajb6b works synergistically with Bag3 protein to regulate proteostasis. Moreover, our F0-based genetic screen also identified *myh9b* as a so-far-unknown modifying gene, a finding that was later confirmed by stable double mutants. *myh9b* encodes a cytoskeletal protein that plays an important role in the initiation step of autophagy (Kruppa et al., 2016). Specifically, Myh9b functions downstream of Ulk1 and Atg8, regulating phagophore formation. Consistent with this notion, we observed further decreased levels of microtubule-associated protein 1A/1B-light chain 3 (LC3), a molecular marker of autophagy, in the *bag3;myh9b* double mutant comparing to the bag3 single mutant (Fig. 7E). Intriguingly, we found mTOR to be a salutary modifier (see above), which functions upstream of Ulk1 to govern the initiation of autophagy (Suvorova and Pospelov, 2019). Our data suggest that *bag3*-mediated cardiomyopathy is characterized by high sensitivity to genetic lesions in the *mtor-myh9b* signaling axis that affects autophagy initiation, a key step of proteostasis. Further studies are, therefore, warranted to elucidate the detailed mechanisms that underlie the modifying effects of *myh9b* on *bag3* cardiomyopathy.

In summary, we proved the feasibility of an F0-based genetic assay in adult fish, which possesses much higher throughput than classic genetic interaction studies that use stable double mutants. As suggested by our pilot screen, the assay is particularly useful in experimentally testing vast number of candidate genes suggested by system biology approaches, such as differentially expressed genes identified from transcriptome analysis. Thus, we anticipate that our assay will help to facilitate the systematic discovery of genetic modifiers for inherited cardiomyopathies. In principle, this F0-based genetic assay should be applicable to other human diseases that can be modeled in the zebrafish.

## MATERIALS AND METHODS

### Animals

Zebrafish (*Danio rerio*) were maintained under a 14 h light–10 h dark cycle at 28.5°C and handled with care. Animal study protocols were approved by the Mayo Clinic Institutional Animal Care and Use Committee (A3513). All animal study procedures were performed in accordance with the Guide for the Care and Use of Laboratory Animals published by the US National Institutes of Health (NIH Publication No. 85-23, revised 1996). The *bag3^e2/e2^* mutants containing 10-nucleotides deletion in the 2nd exon have been reported previously (Ding et al., 2019). Genotyping to discern the *bag3* mutant from WT siblings was carried out by PCR followed by digestion with restriction enzyme PstI. The gene-break transposon (*GBT*) mutants *002/sorbs2b, GBT136/ano5a, GBT411/dnajb6b* and *GBT419/rxraa* have been previously generated and reported (Ding et al., 2013, 2016), and the mutant alleles were identified through genotyping PCR by using gene-specific primer pairs flanking the GBT transposon integration site combined with a RP2 insertional vector-specific primer RP2-5′-LTR or RP2-3′-LTR (Ding et al., 2013). Primer sequences for genotyping PCR are listed in Table S1.

### *In vivo* echocardiography for adult fish hearts

The Vevo 3100 high-frequency imaging system equipped with a 50 MHz (MX700) linear array transducer (FUJIFILM VisualSonics Inc) was used to measure cardiac function indices in adult zebrafish according to reported protocol (Zhang et al., 2018). Briefly, acoustic gel (Aquasonic^®^ 100, Parker Laboratories, Inc) was applied over the surface of the transducer to provide adequate coupling with the tissue interface. Adult zebrafish at appropriate ages were anesthetized in tricaine (0.02%) for 5 min and placed ventral side up into a sponge. The MX700 transducer was placed above the zebrafish to provide a sagittal imaging plane for the heart. B-mode images were acquired by using an imaging field of view of 9.00 mm in the axial direction and 5.73 mm in the lateral direction, a frame rate of 123 Hz, with medium persistence and a transmit focus at the center of the heart. Image quantification was performed using the VevoLAB workstation. Cardiac function indices of the ejection fraction (EF) were calculated using EF=(EDV−ESV)/EDV; fractional shortening (FS) was calculated using FS=(Ld−Ls)/Ld; EDV and ESV are the ventricular volume at the end-diastolic stage and end-systolic stage, respectively. Ventricular chamber dimensions were measured from B-mode images by using the following two indices: EDV/body weight (BW) and ESV/BW (Zhang et al., 2018). Ld and Ls represent the length of the short axis of the ventricle at the end-diastolic stage and end-systolic stage, respectively. For each index on individual fish, measurements were performed on three independent cardiac cycles to acquire average values.

### Evaluation of embryonic heart function

Quantification of zebrafish embryonic heart function was performed using previously reported protocols (Hoage et al., 2012). Briefly, fish embryos at designated stages were anesthetized with tricaine (0.02%) (Argent Chemical Laboratories) for 2 min, placed lateral side up and held in place with 3% methyl cellulose (Sigma-Aldrich). The beating hearts were documented by using a Zeiss Axioplan 2 microscope with a differential interference camera lens. FS was analyzed by Image J and calculated using the FS=(Ld−Ls)/Ld, in which Ld and Ls represent the length of the short axis of the ventricle at the end-diastolic stage and of the end-systolic stage, respectively.

### Measurement of ventricular surface area to body weight ratio

Measurement of ventricular surface area (VSA) to body weight (BW) ratio was performed according to a previously published method (Ding et al., 2011). To measure their body weight, fish were anesthetized in 0.16-mg/ml tricaine solution, semi-dried on a paper towel and weighted. To measure the VSA, ventricles of individual zebrafish were dissected and imaged next to a ruler comprising a scale in mm under a Leica MZ FLI III microscope. The largest projection of a ventricle was outlined using ImageJ software. The VSA/BW index was then calculated by using the largest VSA (in mm^2^) divided by BW (in g).

### Histology and transmission electron microscopy

For hematoxylin and eosin (H&E) staining, heart tissues were dissected from individual adult fish at the designed stages after euthanasia by incubation with 0.032% tricaine for 10 min. Isolated hearts were immediately fixed in 4% PBS-buffered formaldehyde and sent to the Mayo Clinic Histology Core Laboratory for sample processing and H&E staining. Images of the apex region were captured by using the EVOS FL Auto Imaging System (ThermoFisher Scientific). The density of the trabecular muscle was quantified by using ImageJ software. For the transmission electron microscopy (TEM) study, adult zebrafish hearts at designated stages were dissected and immediately fixed in fixation solution (4% paraformaldehyde and 1% glutaraldehyde in 0.1 M phosphate buffer pH 7.2) at room temperature for 1 h, followed by overnight incubation at 4°C. Fixed samples were subsequently processed and imaged at the Mayo Clinic Electron Microscopy Core Facility using a Philips CM10 transmission electron microscope.

### Western blotting

Individual fish embryos at 1-3 days post fertilization (dpf) (Fig. 3C) or individually isolated adult fish heart ventricles (Fig. 2E, 3E and 4G) were transferred to the RIPA lysis buffer (Sigma-Aldrich) supplemented with complete protease inhibitor cocktail (Roche) and homogenized using a Bullet Blender tissue homogenizer (Next Advance, Inc). Approximately 1-5 µg resultant protein lysate was subjected to western blotting following a standard protocol. The following primary antibodies were used: anti-GAPDH (1:5000; Santa Cruz Biotechnology Inc., catalog #sc-25778), anti-mTOR (1:3000; Cell Signaling Technology, catalog #2983), anti-ubiquitin (1:1000; ThermoFisher Scientific, catalog #PA5-17067) and anti-LC3 (1:3000; Cell Signaling Technology, catalog #12741). All these antibodies have previously been demonstrated to work in zebrafish (Bu et al., 2021; Ding et al., 2019).

### Predicted MMEJ-based sgRNA design and F0 injection

Sequences for genes of interest were acquired and downloaded from Ensembl (http://useast.ensembl.org/index.html). Preferred exons located toward the N-terminal were then uploaded to MENTHU (http://genesculpt.org/menthu/) for guide RNA target selection. Target sequences with scores >1.0 were chosen from predicted MMEJ loci (Table 1). Single guide RNAs (sgRNAs) with appropriate chemical modifications were synthesized and obtained from the Synthego (Synthego Corporation). sgRNAs were dissolved in nuclease-free duplex buffer (Integrated DNA Technologies, catalog #11-01-03-01) to obtain a 100 µM concentration as stock solutions and further diluted to 2 µM to 10 µM as working concentrations.

Alt-R Cas9 protein (Integrated DNA Technologies, catalog #1081058) was diluted to a working concentration of 3.3 µg/µl in buffer (20 mM HEPES, 100 mM NaCl, 5 mM MgCl_2_, 0.1 mM EDTA pH 7.5). Working concentrations of sgRNA and Cas9 were then mixed at a ratio of 1:1 (1.5 µl of gRNA+1.5 µl of Cas9 protein) and incubated in a water bath at 37°C for 10 min to assemble the sgRNA-Cas9 protein (sgRNP) complex. Approximately 1 nl of the sgRNP complex together with 0.01% Phenol Red was then injected to one-cell-stage fish embryos to obtain the predicted MMEJ-injected F0 embryos. For each predicted MMEJ-inducing sgRNA, we tested different injection doses and usually measured the KO score in eight injected embryos, aiming to identify sgRNAs that result in >50% KO score within all embryos injected. After the injected embryos reached adult stage, we confirmed the efficiency of sgRNAs by measuring KO scores again by performing genotyping PCR, using genomic DNA isolated from tail fins. Phenotyping analysis was carried out on all animals injected with the same sgRNA and by deprioritizing sgRNAs with lower KO scores.

### Calculation of KO scores

Collected were either individual predicted MMEJ sgRNPs-injected embryos or tail fins from adult fish. Samples were transferred into 50 µl alkaline lysis buffer (25 mM NaOH, 0.2 mM EDTA pH 12) and incubated for 30 min at 95°C, followed by addition of 50 µl neutralization buffer (40 mM Tris-HCl pH 5.0). Resultant genomic DNA lysates (1 µl) were used as templates for PCR analysis to quantify the KO score for each predicted MMEJ sgRNA. The PCR primer sequence information for KO score quantification is listed in Table S3. To quantify the score, 5 µl PCR amplicon was digested with Exo I and sent for Sanger sequencing at Genewiz (https://clims4.genewiz.com/CustomerHome/Index). Chromatograms from two PCR amplicons, using either predicted MMEJ sgRNA-injected or non-injected embryonic genomic DNA lysates as templates, were analyzed for KO score calculation by using the R code-based open access software Inference of CRISPR Edits (ICE) v2 CRISPR Analysis Tool by TIDE (https://www.synthego.com/products/bioinformatics/crispr-analysis) (Brinkman and van Steensel, 2019). Microhomology alleles (in %) were calculated by dividing the KO score for the predicted microhomology indel by the total KO score.

### Real-time quantitative reverse transcription PCR

Total RNA was extracted from a zebrafish larva or an individual adult fish ventricle by using Trizol (ThermoFisher Scientific) following the manufacturer's instruction. Approximately 100 ng total RNA was used for reverse transcription (RT) and cDNA synthesis by using the Superscript III First-Strand Synthesis System (ThermoFisher Scientific). Real-time quantitative RT-PCR (real-time qRT-PCR) was carried out in 96-well optical plates (ThermoFisher Scientific) using Applied Biosystem VAii 7 System (ThermoFisher Scientific). Gene expression levels were normalized using the expression levels of glyceraldehyde 3-phosphate dehydrogenase (*gapdh*) or actin, beta 2 (*actb2*) by –ΔΔCt (cycle threshold) values. All quantitative RT-PCR primer sequences are listed in Table S2.

### Co-immunoprecipitation assays

Human DNAJB6 cDNA was synthesized by RT-PCR using total RNA isolated from normal human adult heart (Biochain) and cloned into a pShuttle plasmid in-frame with the FLAG tag (Addgene), resulting in generation of the pShuttle-DNAJB6-FLAG construct. Human BAG3 cDNA was synthesized from total RNA isolated from normal human adult heart and cloned into the pShuttle plasmid in-frame with the HA tag (Addgene), resulting in generation of the pShuttle-BAG3-HA construct. 1 µg of either single or double constructs were transfected into HEK293 cell kept at ∼80% confluency within a p10 dish. Co-immunoprecipitation experiments were carried out using the FLAG^®^ Immunoprecipitation Kit (FLAGIPT1, Sigma) according to the manufacturer’s instructions. Anti-FLAG and anti-HA antibodies (both Sigma) were used at dilutions of 1:10,000.

### Statistics

Sample sizes were never calculated before performing the experiments. No animals were excluded for analysis. Unpaired two-tailed Student's *t*-test was used to compare two groups. One-way analysis of variance (ANOVA) was used to assess differences between three or more groups. To compare animal survival rates, log-rank test was used to determine differences. Survival curves are cumulative results of fish from several breeding efforts. For all dot plots, each value represents mean±standard error (±s.e.). Sample size (*n*) indicates number of animals. All statistical analyses were performed with the GraphPad Prism 7. For post hoc analysis, Tukey's test was employed to confirm our findings.

## Supplementary Material

Supplementary information
