# Supplementary material for: Application of an F0-based genetic assay in adult zebrafish to identify modifier genes of an inherited cardiomyopathy
Source: Dis Model Mech. 2022 Jun 23;16(5):dmm049427. doi: 10.1242/dmm.049427 (PMC9239171; doi:10.1242/dmm.049427)
Supplement: Supplementary information [file dmm-16-049427-s1.pdf]

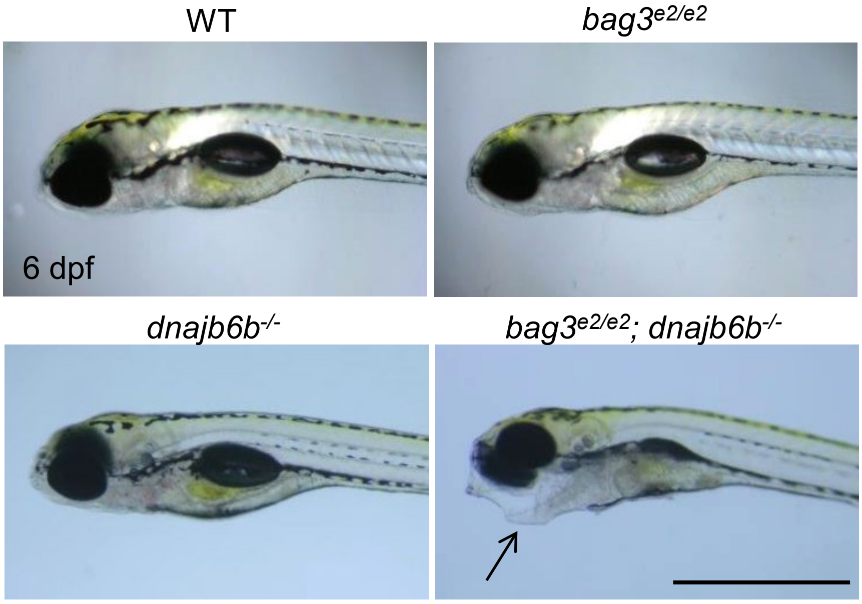


**Fig. S1. The homozygous *GBT411/dnajb6b* mutant exerted deleterious effects on the *bag3$^{e2/e2}$* mutant**

Images of the *bag3$^{e2/e2}$; dnajb6b$^{-/-}$* double mutant fish compared to the *bag3$^{e2/e2}$* or *dnajb6b$^{-/-}$* single mutants and WT controls at 6 days post-fertilization (dpf). Scale bar, 1 mm.

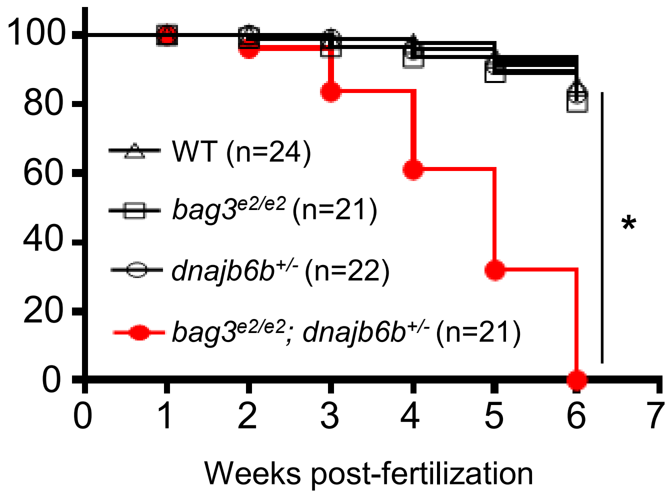


**Fig. S2. The heterozygous G*BT411/dnajb6b* mutant exerted deleterious effects in survival on the *bag3*$^{e2/e2}$ mutant**

Survival curves of *bag3*$^{e2/e2}$*; dnajb6b*$^{+/-}$ double mutant fish compared to single mutants and WT controls. * N=21-24. * $P$<0.05, Log rank test.

Disease Models & Mechanisms • Supplementary information

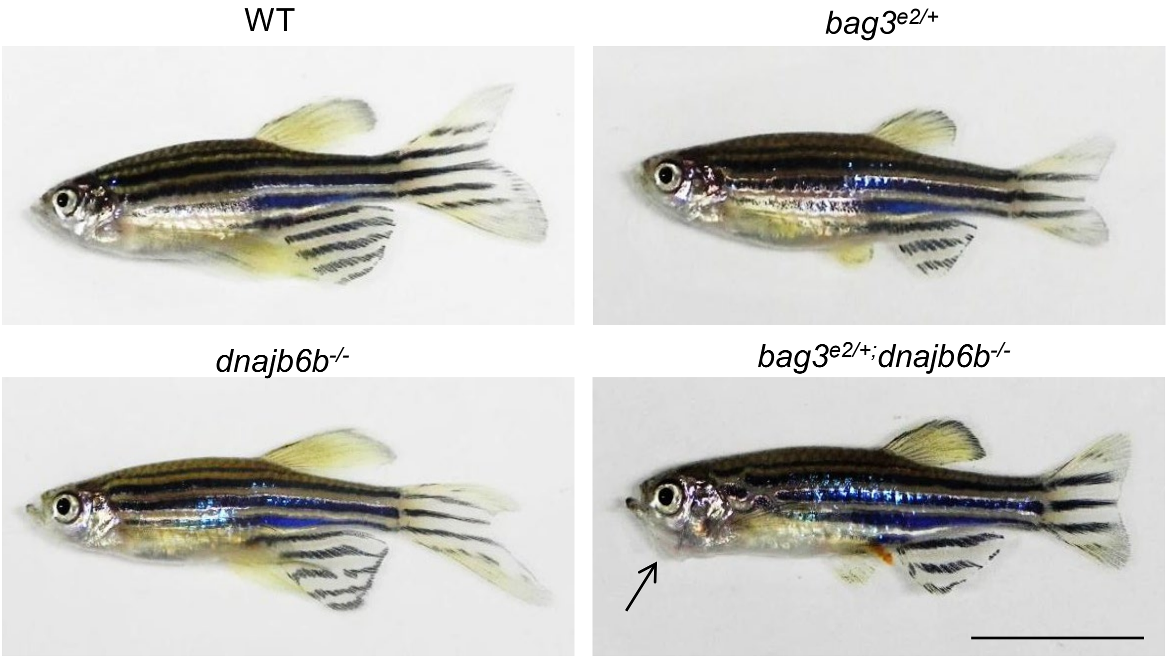


**Fig. S3. Genetic interaction between *bag3*$^{e2/+}$ and *dnajb6b*$^{-/-}$ mutant fish** Representative images of *bag3*$^{e2/+}$; *dnajb6b*$^{-/-}$ double mutant fish and their corresponding single mutant controls at 3 months of age. The arrow points to unusual jaw protruding phenotype. Scale bar: 1 cm.

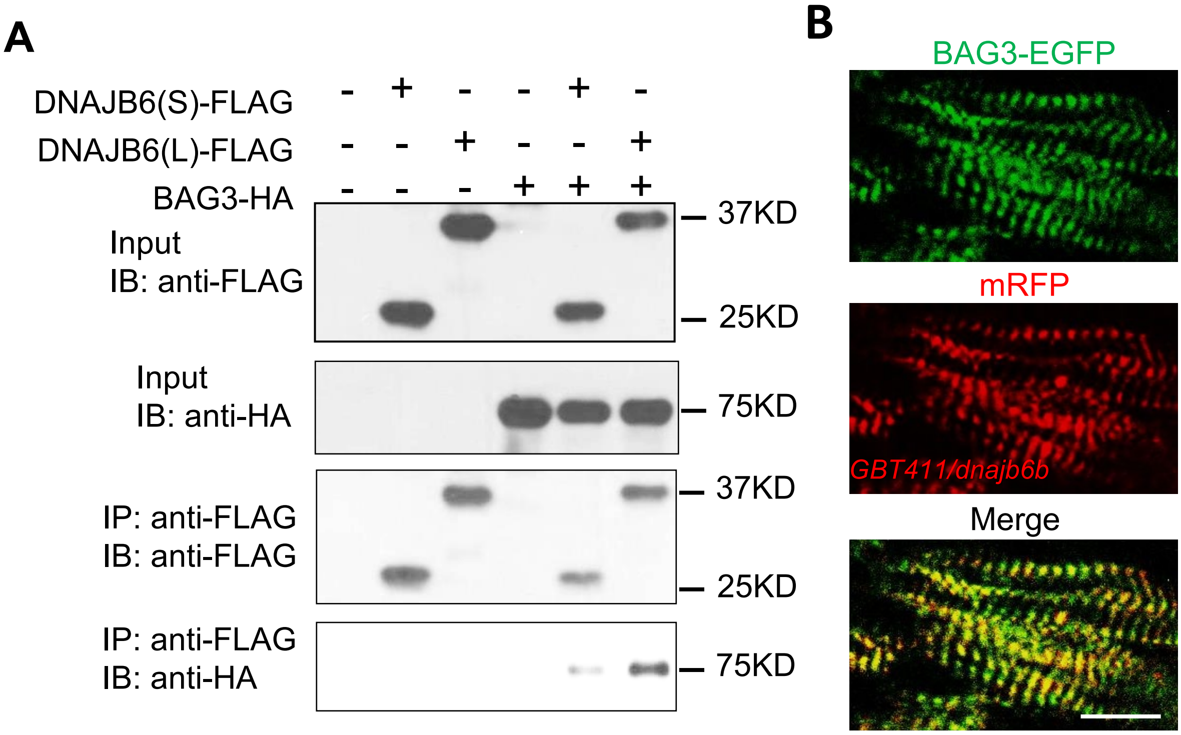


**Fig. S4. DNAJB6 protein interacts with BAG3 *in vitro***

**A,** Western blot analysis of the *in vitro* protein pull-down assay indicated a direct protein-protein interaction between the human DNAJB6(L) and BAG3 proteins and between the human DNAJB6(S) and BAG3 proteins in the HEK293 cells. IP: immunoprecipitation. IB: immunoblot. **B**, Fluorescent images of adult heart sections from *GBT411/dnajb6b* mutant after crossed with the *Tg(cmlc2:BAG3-EGFP*) transgenic fish. Scale bar, 10 μm.

Disease Models & Mechanisms • Supplementary information

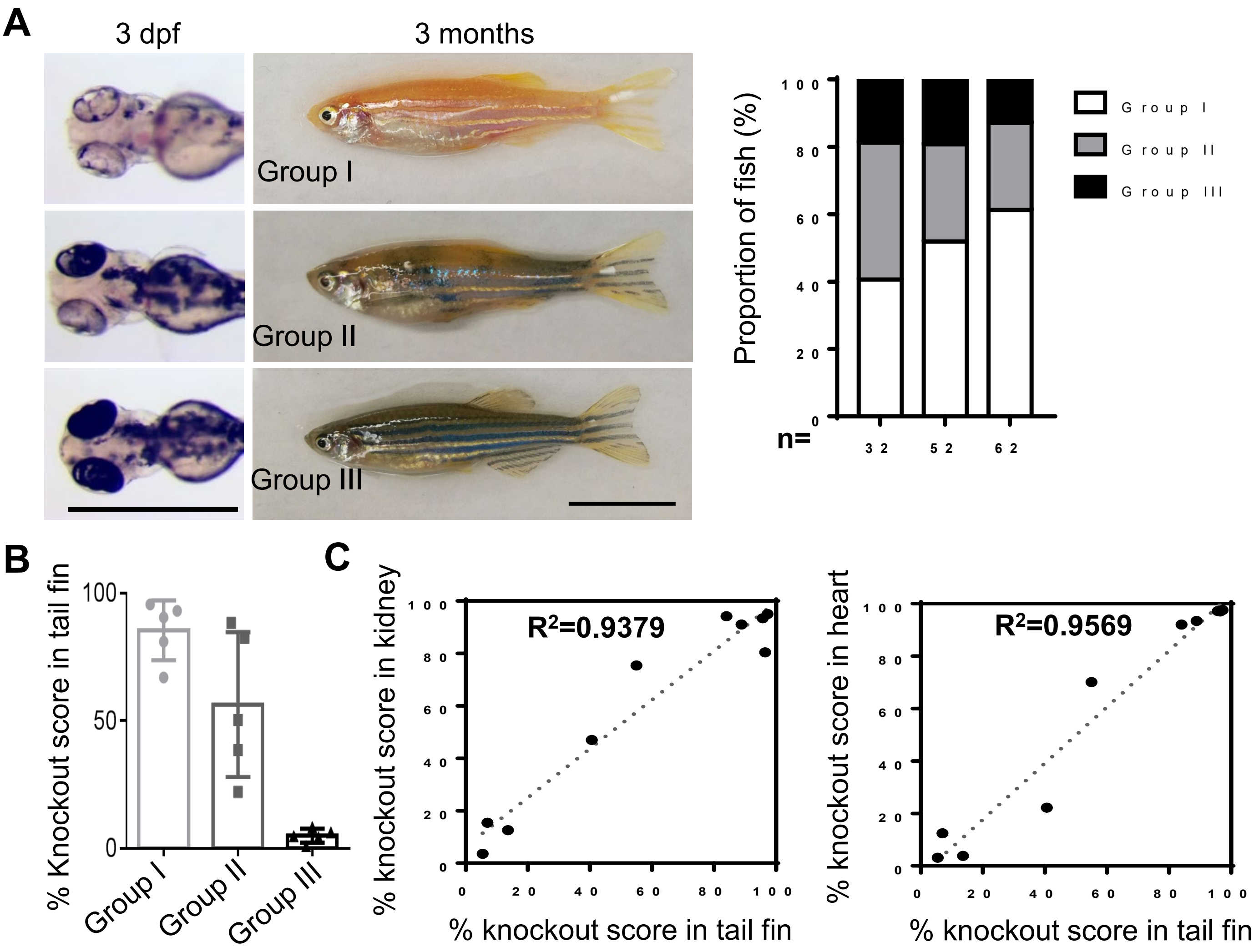


**Fig. S5. Effective gene knockout by microhomology-mediated end-joining (MMEJ)-based *tyrosin* single guide RNA sustained from embryonic to adult stages**

**A-B,** Representative images and quantification of *tyrosine* (*tyr*) microhomology-mediated end-joining (MMEJ)-based single guide RNA (sgRNA) injected fish at 3 dpf and 3 months categorized from high to low knockout (KO) score (group I to group III). Scale bar in left panels, 1mm, right panels, 1 cm. Three bars in the bar graph in (**A**) represent results from three independent injections of *tyr* sgRNA. **C,** Correlation of KD score calculated using genomic DNA extracted from the tail fin, heart or kidney, respectively.

Disease Models & Mechanisms • Supplementary information

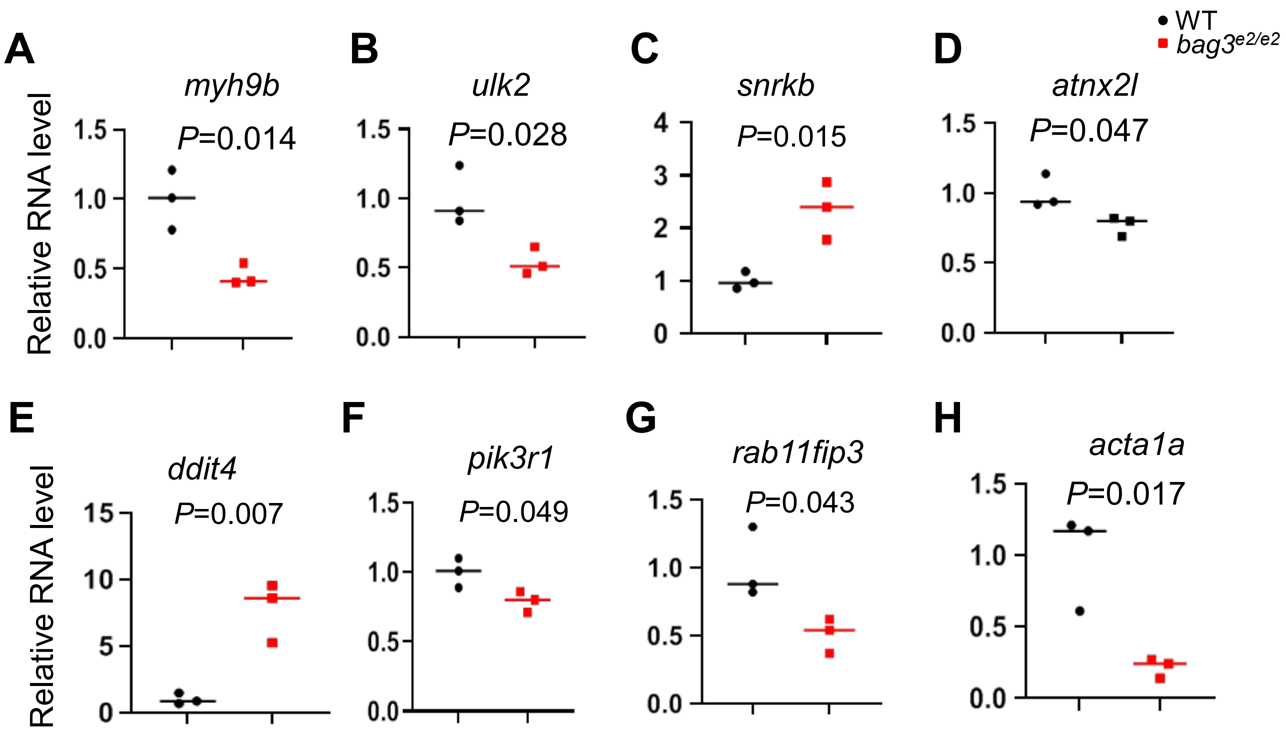


**Fig. S6. Quantitative RT-PCR results of 8 differentially expressed proteostasis/autophagy genes in the *bag*$^{e2/e2}$ cardiomyopathy model**

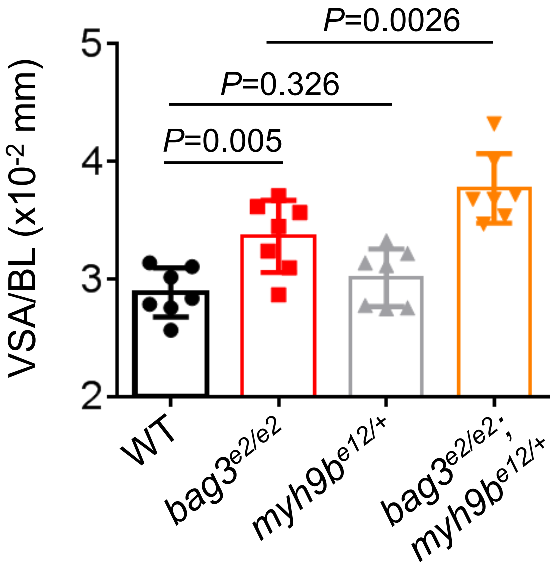


**Fig. S7. *The bag3$^{e2/e2}$;myh9b$^{e12/+}$* double mutant fish displayed enlarged ventricular chamber size**

Quantification of the ventricular surface area (VSA) normalized to the body length (BL) of fish at 6 months. N=6, one-way ANOVA.

Disease Models & Mechanisms • Supplementary information

**Table S1.** Primers for genotyping PCR to identify the *bag3*$^{e2/e2}$ and gene-break transposon (GBT) mutants

| Primer name | Primer Sequence |
|---|---|
| RP2-5’-LTR | 5’-GCATGAACTCCTTGATGACG-3’ |
| RP2-3’-LTR | 5’-GTACAGTAATCAAGTAAAATTACTCA-3’ |
| GBT002/sorbs2b-F | 5’-GGGTCGGCATGCTTCATCACTG-3’ |
| GBT002/sorbs2b-R | 5’-AATCCAGCAGACCGAACAAACGC-3’ |
| GBT136/ano5a-F | 5’-AGGGTCTTTGTGGATGTCTGTGAATG-3’ |
| GBT136/ano5a-R | 5’-CTGAGGTCTCTGCACAACCTATTAGTGT-3’ |
| GBT411/dnajb6b-F | 5’-TGTGATAACATCACAGCACC-3’ |
| GBT411/dnajb6b-R | 5’-ACGATGAAAATGAGCTGAAGC-3’ |
| GBT419/rxraa-F | 5’-ATATCGCTTTAGTTCGCTCACTTTAT-3’ |
| GBT419/rxraa-R | 5’-TATATACACGAAAATCCTCCACACA-3’ |
| bag3-F | 5′-CGGCGTATAAAGAATTGCTGG-3’ |
| bag3-R | 5′-GTGAAGTAGGTGAGCAAGAC-3’ |

Disease Models & Mechanisms • Supplementary information

**Table S2.** Quantitative RT-PCR primers used to validate differentially expressed (DE) genes

| Gene | Primer Sequence |
|---|---|
| *acta1a* | F: 5’-CAGCTTTACTTCAGGACTCACG-3’ |
| | R: 5’-TACCTCTCTTGCTCTGAGCCTC-3’ |
| *ddit4* | F: 5’-AGCCATCAGACTTGAGGATAC-3’ |
| | R: 5’-GGGTCGAAAAACTCAGATTGG-3’ |
| *atnx2l* | F: 5’-ACCCGAGACACTTTCACAG-3’ |
| | R: 5’-GACTGGAGTCATAGGTGGAC-3’ |
| *pik3r1* | F: 5’-AACTCCTAAACCGAGACCTC-3’ |
| | R: 5’-AGCACTGTCTGACATCCAG-3’ |
| *snrkb* | F: 5’-CGG ACGCATGAAGATTTCAG-3’ |
| | R: 5’- CATATAGACCAGCTATCTTGCC-3’ |
| *ulk2* | F: 5’-AAGACTGATTGGGAGGTCG-3’ |
| | R: 5’- GCTGCAATCTGCTGTAGAAAG-3’ |
| *myh9b* | F: 5’-ATCTACTCAGAGGAGATCGTG-3’ |
| | R: 5’- GCTGCTCTGGTCTTTCTTG-3’ |
| *rab11fip3* | F: 5’-AGGCATCAGTGCAATCAGC-3’ |
| | R: 5’-GTCGTCTCCATCTGTGAGT-3’ |

Disease Models & Mechanisms • Supplementary information

**Table S3.** PCR primers for quantifying knockout efficiency of predicted MMEJ-inducing sgRNAs

| Gene | Primer Sequence |
|---|---|
| *atxn2l* | F: 5'-CCTGCCTAATCGAACAAATCG-3' |
| | R: 5'-GTGTGAGAGTGAATGTGAGAG-3' |
| *myh9b* | F: 5'-GAATGTGACCGATTTCACCC-3' |
| | R: 5'-CCTCAAAGATCTCAAAGCCG-3' |
| *ulk2* | F: 5'-CTGAAGGCTGTCTCAGATTG-3' |
| | R: 5'-ACTTTAGGCATTCAACCGTAG-3' |
| *snrkb* | F: 5'-CGGACGCATGAAGATTTCAG-3' |
| | R: 5'-CATATAGACCAGCTATCTTGCC-3' |
| *rab11fip3* | F: 5'-AGGCATCAGTGCAATCAGC-3' |
| | R: 5'-GTCGTCTCCATCTGTGAGTG-3' |
| *pik3r1* | F: 5'-AACTCCTAAACCGAGACCTC-3' |
| | R: 5'-AGCACTGTCTGACATCCAG-3' |
| *ddit4* | F: 5'-GATAACTGATCATGCAGGACTG-3' |
| | R: 5'-TGCACACCTGAATCTGAGG-3' |
| *acta1a* | F: 5'-CACCACACCTTCTACAATGAG-3' |
| | R: 5'-ACATCAGAGTTTTCGATCAGC-3' |
| *dnajb6b* | F: 5'-CACAGTGAGCCTGAAATGATG-3' |
| | R: 5'-AGCTGCACACAGATTAACATAC-3' |
| *mtor* | F: 5'-AGGTGCAGCCATTCTTTGAT-3' |
| | R: 5'-ATGTCATACCTCTCCCTCCATAC-3' |
| *tryosinase* | F: 5'-GCGTCTCACTCTCCTCGACTCTTC-3' |
| | R: 5'-GTAGTTTCCGGCGCACTGGCAG-3' |

Disease Models & Mechanisms • Supplementary information
